# Brain-wide imaging of neurons in action

**DOI:** 10.3389/fncir.2014.00031

**Published:** 2014-04-03

**Authors:** Erick T. Tatro

**Affiliations:** Department of Psychiatry, University of California San DiegoLa Jolla, CA, USA

**Keywords:** whole brain imaging, *C. elegans*, *R. danio*, calcium sensor, two-photon imaging, GFP CalModulin proteins, light-sheet imaging, sculpted light imaging

Fully comprehending the brain as an information-processing organ will involve analysis of both the physical and algorithmic components of the system. The physical substrates comprising the brain, including neurophysiology, ion gradients, neurotransmitter release, and the molecules involved in action potential firing have been studied since Hodgkin and Huxley developed the squid giant axon model in 1952 Hodgkin et al. (1952). In order to understand how complex properties like response to stimulus, cognition, and emotion are derived from simple physical processes like an action potential, the algorithms of neural circuitry need to be described (Power et al., 2011). Mapping whole brain neural networks (Figure 1) and describing dynamic interactions during behavior is a daunting technological challenge that neuroimaging researchers are tackling with greater precision. In the March and September 2013 issues of Nature Methods, Ahrens et al. (2013) and Schrödel et al. (2013) describe whole-brain functional imaging at the single-neuron level in two model organisms adapting two fluorescence microscopy techniques.

**Figure 1:**
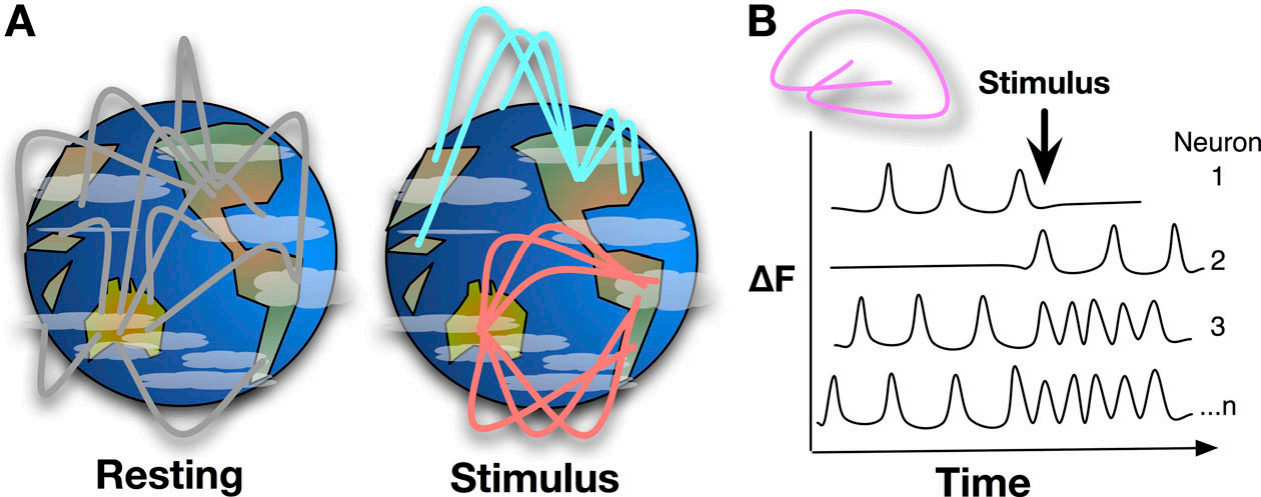
**(A)** Mapping whole brain neural activity is a daunting task, it involves determining how interacting networks respond to stimuli, encode information, and change with development, experience, and aging. Global network interactions of the brain can be described like linkages across continents, with background signaling occurring during resting state, e.g., asynchronous networks as described in Ahrens et al. Upon stimulation, a pathway or node may be activated while suppressing others, changing the global signaling network during and shortly after stimulation. Whole-brain, single-cell resolution, monitoring of activity over time will help to map these networks and nodes. **(B)** Detecting Ca^2+^ bursts via fluorescence (δF) in individual neurons brain-wide shows how neuron actions are correlated and anti-correlated at resting state or during stimulus and behavior.

At the core of both reports is the use of genetically encoded Ca^2+^ reporters that fluoresce in the presence of high Ca^2+^ concentration. During an action potential of a neuron, Ca^2+^ is released into the cytoplasm and the Ca^2+^ is quickly returned to its compartments in the endoplasmic reticulum and extracellular space as the neuron restores resting potential. Thus a Ca^2+^ “bursts” marks a firing neuron. The genetically encoded reporters are derived from fusing the Ca^2+^-binding domain from calmodulin, another peptide domain, and a circularly permuted green fluorescent protein (GFP) whose fluorescence properties are modulated by the Ca^2+^ sensing interaction. These are termed GFP CalModulin proteins (GCaMP), which have been modified and optimized for Ca^2+^ responsiveness and fluorescence properties (Akerboom et al., 2012). While the GCaMPs have been used since 2001 for detecting Ca^2+^ activity in the roundworm (Nakai et al., 2001; Chen et al., 2013), *Caenorhabditis elegans*, Schrödel et al. (2013) developed a sculpted light technique for recording activity of the majority of head ganglion neurons in response to chemical stimulus with high temporal and spatial resolution. Ahrens et al. (2013) used laser scanning light-sheet microscopy of the albino zebrafish, species *Danio rerio*, to capture activity of over 80% of the neurons in the brain every 1.3 seconds and identified anticorrelated hindbrain oscillations (Ahrens et al., 2013).

The light-sculpted technique involves two-photon excitation in which the spectrum of a femtosecond laser pulse is temporally separated by a grating. Thus, the light is separated into its component wavelengths (hence, “sculpted”), and pulsed, rather than continuously illuminated, to the sample in such a manner that the spectral components of the pulse overlap in time and space only at the focus region with near diffraction-limited axial (i.e., z-plane) confinement. With this technique, they achieved a wide-field excitation area of 60 μm diameter with 1.9 μm axial confinement. Imaging a depth of 30 μm, they were able to essentially detect neuronal Ca^2+^ pulses in the entire volume of an 85,000 μm^3^ cylinder of the *C. elegans* brain 4–6 times per second. By further adapting the GCaMP with a nuclear localization signal, they were able to increase resolution to single-cell without sacrificing the ability to detect fast Ca^2+^ spikes.

The light-sheet microscopy technique described in Ahrens et al. (2013) allows for larger volume of detection in the zebrafish brain but with diminished time resolution. Using a laser to scan a 4 μm excitation beam vertically across the head of a live zebrafish, and the objective capturing emitted fluorescence from the active neurons. In 5 μm steps, with 30 ms scans per section, the Ca^2+^ dynamics of a 9.6 × 10^7^ μm^3^ box is captured in 1.6 s.

Both technologies have the ability to nearly simultaneously detect neuronal firing across the whole brain of a model organism using genetically encoded, neuronally expressed, calcium detectors. The advantage of *C. elegans* and the sculpted light technique is the ability to demonstrate neuronal response to chemical stimulus with high time resolution and that all 302 neurons and 8000 synaptic connections have been mapped (White et al., 1986). On the other hand, the light-sheet technique records a larger volume and two orders of magnitude more neurons without the necessity of nuclear localized GCaMP to achieve single-cell resolution. Computational methods to analyze the activity of a large number of neurons (100,000 for the zebrafish) are being developed to derive meaning from these studies. Ahrens et al. (2013) did show anticorrelated oscillations during resting state. With modification and specialization of the GCaMP genetics, it will be possible to describe the functional networks involved in resting state activity, stimulus, information integration, and behavior. GCaMP expression limited to specific neural types like inhibitory, excitatory, or neurotransmitter-specific cells will begin to identify how regulatory networks and activation pathways are encoded in the brain architecture.

Using these techniques to probe behavior will help to establish basic principles of neural circuit function. They may also be used to analyze activity-dependent growth and function across the brain, or brain-wide neuronal activity through development and aging. Understanding how the activity of neural circuits are altered when exposed to drugs of abuse will also help us understand the dynamic processes of addiction and habit. We are beginning to understand that behavior, consciousness, and information processing function of the brain are derived from the retrieval and integration of information encoded in neurons and describing the network properties as they happen is an important first-step in mapping the brain. As future work is prompted by the international efforts of Brain Research through Advancing Innovative Neurotechnologies (BRAIN) Initiative (Insel et al., 2013), announced in April 2013, we should expect variations on these technologies to begin decoding algorithms of the complex network properties of brain function and mental health.

Erick T. Tatro is supported by the US National Institutes of Health grants R01MH94159, R03DA033849, P50DA026306, and R21DA036423.

## Conflict of interest statement

The author declares that the research was conducted in the absence of any commercial or financial relationships that could be construed as a potential conflict of interest.
